# Making sense of complexity: Advances in bioinformatics for plant biology

**DOI:** 10.1002/aps3.11538

**Published:** 2023-08-09

**Authors:** Katie Emelianova, Diego Mauricio Riaño‐Pachón, Maria Fernanda Torres Jimenez

**Affiliations:** ^1^ Department of Botany and Biodiversity Research University of Vienna Vienna Austria; ^2^ Center for Nuclear Energy in Agriculture University of São Paulo São Paulo Brazil; ^3^ Life Sciences Center Institute of Biosciences, Vilnius University Vilnius Lithuania

Coined by Dutch theoretical biologists in the 1970s, the term bioinformatics originally denoted a broad concept relating to the study of information processing in biological systems, such as ecosystem interaction, neuronal messaging, and transfer of genetic information (Hogeweg, 2011). Subsequently co‐opted to describe the sequencing and analysis of molecules (from nucleic acids to proteins), bioinformatics has diverse applications including the analysis, visualization, storage, and generation of data relating to living organisms and the molecular information they carry. Plant biology has reaped dividends from the development and maturation of bioinformatics; it has not only extended our understanding of model plant species such as *Arabidopsis thaliana* (Cantó‐Pastor et al., 2021) but also driven innovative solutions to characterize non‐model species (Nevado et al., 2014). Both avenues of discovery contribute to key objectives in improving food security, conservation, and biotechnology.

The size and complexity of many plant genomes has historically made their analysis financially and computationally difficult. Frequent polyploidy and repeat element expansion make the elucidation of plant genome sequences challenging (Soltis et al., 2015). Furthermore, high heterozygosity in wild populations, pervasive hybridization, and a lack of inbred lines present roadblocks to analyses such as read mapping and assembly (Kajitani et al., 2019). Long‐read technologies have become ever more accessible in recent years, and algorithmic advances have accommodated sequential updates to error models, read lengths, and library types (Michael and VanBuren, 2020). Moreover, novel methods to scaffold contigs and obtain long‐range interaction information have driven impressive improvements in genome assembly quality, making telomere‐to‐telomere genome sequencing projects an achievable goal for many labs (Kress et al., 2022).

Long‐read technologies paired with novel mapping algorithms have fueled discovery of new transposable element (TE) dynamics, and there has been an associated resurgence of interest in their role in adaptive trait evolution and phenotypic variation (Schrader and Schmitz, 2019; Pimpinelli and Piacentini, 2020). Bioinformatics developments in this field have led to vast improvements in our ability to detect complex TE mobilization patterns such as nested insertions and structural variants (Bree et al., 2022; Lemay et al., 2022). Despite these advancements, characterization and annotation of genomic features such as genes and repetitive elements remain challenging due to species‐specific genomic configurations, taxonomically patchy reference databases, and a lack of robust benchmarking and quality control. While structural and functional annotation methods still have significant obstacles to overcome, many important contributions have been made to improve the comparison and optimization of these approaches (Caballero and Wegrzyn, 2019). Moreover, the extension and aggregation of existing gene, variant, and repeat annotation software is beginning to allow researchers to combine and curate different algorithmic approaches and databases (Nelson et al., 2017; Kirsche et al., 2023).

The scale of plant diversity to be characterized remains a challenge, however, and incorporating samples from preserved, non‐model, or difficult‐to‐access material requires innovative wet lab and bioinformatics solutions (Lang et al., 2020). Reduced representation sequencing (RRS) methods represent a crucial tool for the study of non‐model plants; this adaptation of emerging sequencing technologies has allowed for cost‐effective population studies, analyses of historical diversity using herbarium specimens, and phylogenomic explorations on a large scale (Kersey, 2019; One Thousand Plant Transcriptomes Initiative, 2019). Limitations associated with RRS such as paralogous genes, different selection landscapes of coding and non‐coding sequences, and missing data are increasingly accounted for with the continuous improvement of software and methodology (Johnson et al., 2016), and integration of ‐omics data for non‐model taxa in online portals creates an ever more accessible environment for researchers to characterize the world's flora (Goodstein et al., 2012).

Bioinformatics, since its inception in biological applications, has been a field in constant flux, with a high turnover of technologies, sequencing platforms, algorithms, and techniques, and the current landscape of bioinformatics in plant sciences is no different. This special issue of *Applications in Plant Sciences* presents five papers that explore bioinformatics approaches to address issues in plant biology, such as genome assembly, reduced representation sequencing, and structural and functional annotation. We summarize these papers here.

Reduced representation sequencing methods such as target capture, RAD sequencing, and genome skimming provide powerful tools for phylogenomic studies, especially in cases where whole genome analyses are infeasible or many non‐model organisms must be sampled cost efficiently. Bioinformatic methods such as probe design and resolution of paralogous sequences have critical impacts on downstream analyses and interpretations; therefore, clear guidelines and accessible implementation are important to ensure that maximum benefits are reaped by the scientific community. Two papers in this issue discuss aspects of RRS.

Despite recent advances in whole genome sequencing, RRS approaches continue to be of great importance in biodiversity and evolutionary studies, particularly in situations where obtaining fresh plant material is not feasible or the number of samples is very large. In their contribution, Pezzini et al. (2023) provide a comprehensive review of genome skimming and target capture, two techniques used commonly for the study of non‐model organisms and difficult material such as herbarium specimens. This review is timely, because while the design of target capture probes (i.e., bait sets) for specific taxa has historically been hindered by the limited availability of genomic resources for non‐model organisms, this is likely to change in the next few years thanks to ambitious whole genome sequencing efforts such as the Earth Biogenome Project (Lewin et al., 2022). The rapid growth in the number and taxonomic resolution of bait sets is making analysis of non‐model plant species easier by using probes that are universal or cover larger clades. Pezzini and co‐authors discuss a variety of approaches utilizing existing resources such as combining universal and taxon‐specific bait sets for use in non‐model organisms, or combining new results with legacy data to enable broader taxon sampling. Considerations for genome skimming and target capture have similarities; however, the untargeted technique used by genome skimming results in sequence data that are highly dependent on copy number, favoring more frequently represented regions such as those in chloroplasts and mitochondria. Including both project planning and downstream analysis considerations, the authors review the merits and drawbacks of both target capture and genome skimming approaches, providing a valuable resource for researchers who may have a variety of data, taxa, and tissue types at hand.

In their contribution to this issue, Jackson et al. (2023) build on the existing bioinformatic pipelines HybPiper (Johnson et al., 2016) and ParaGone (Yang and Smith, 2014), providing a streamlined version of both pipelines within a Singularity container, vastly simplifying dependency installation and implementation. These two pipelines perform target capture read assembly and paralogy resolution, respectively, and the use of both is a common workflow employed by phylogeneticists prior to species tree inference. Within the containerized pipeline, the authors implement two Nextflow workflows, hybpiper‐nf and paragone‐nf, which include improved sample handling and methodological improvements. Hybpiper‐nf addresses organization and tractability of large sample sizes, automatically detecting sequence types in BLAST (Altschul et al., 1990) and Diamond (Buchfink et al., 2015) runs and parsing sequence names from read files. Additional improvements over the previous standalone implementations of HybPiper include additional options to manipulate the resolution of chimeric locus assemblies, giving the user greater insight and control over the processing of target capture data. The process of phylogenomic inference is streamlined by the production of correctly formatted files from hybpiper‐nf that are directly compatible with paragone‐nf, where four different paralog inference algorithms are implemented (originally described in Yang and Smith [2014]). The authors test their workflow using the Angiosperms353 and Compositae1061 bait sets applied to data sets including Asteraceae and Orchidaceae, demonstrating greatly improved usability and streamlining of the target capture workflow. This new, containerized workflow will provide the non‐model plant biology community with more accessible bioinformatic tools to analyze RRS data and greatly streamline new phylogenomic projects.

Transposable elements are a ubiquitous feature of plant genomes, and the revival of interest in TEs and their role in genome dynamics, trait evolution, and evolutionary trajectories has coincided with the emergence of long‐read sequencing technologies, which can allow researchers to capture 5′ and 3′ insertion sites in a single read, a feat not previously possible with short reads. Popular TE annotation software, however, remains computationally inaccessible for some researchers due to long run times and high computational demands. Gonzalez‐García et al. (2023) leverage algorithmic advances in long‐read mapping techniques to annotate TEs, using a computationally efficient homology‐based method employing minimizers. The comparatively high error rate of long reads is a useful proxy for the imperfect sequence conservation between members of TE families, and the authors build on the long‐read alignment method used by Minimap2 (Li, 2018) to reduce run time from hours to minutes, marking an improvement of orders of magnitude in computational efficiency. Moreover, the authors make use of alternatives to commonly used de novo TE annotation pipelines (Orozco‐Arias et al., 2023), broadening the diversity of bioinformatic resources for TE annotation, a field which, despite its age, still presents significant challenges in model and non‐model organisms alike.

The annotation of gene features is a fundamental step in ascribing context to genomic data sets, paving the way for further studies such as expression assays, comparative genomics, and population dynamics. Despite advances in genome assembly methods, genome annotation remains one of the most challenging bottlenecks facing plant genome science, with intron length variation, divergent TE dynamics, and low sequence conservation hampering the annotation efforts of non‐model genome projects. In their contribution, Vuruptoor et al. (2023) address the need to improve quantification of structural genome annotation methods, employing a mixture of existing and emerging metrics to benchmark genome annotation methods. They approach the issue in a robust manner by using a broad diversity of taxa with challenging genomic features such as variable ploidy, high TE content, and large genomes. As well as commonly used metrics such as BUSCO, the authors draw attention to equally informative measures of annotation quality such as the ratio of mono‐exonic to multi‐exonic genes to detect unlikely gene models and false positive genes resulting from incomplete repeat masking. That the problem of genome annotation is not solved, even in model plant species, is testament to the importance of benchmarking studies such as this, and the inclusion of challenging taxa during software design is vital to ensure non‐model plant species can equally benefit from bioinformatic innovations.

Upstream bioinformatics analyses frequently produce an extensive list of genes of interest, for example, transcripts that are differentially expressed between control and perturbed conditions, genes that show signals of accelerated rates of evolution, or particularly duplication‐rich gene families. In order to make these results statistically meaningful and human readable, further contextualization is required through categorizing the genes employing the widely used system of gene ontology (GO). In GO, hierarchical structures of molecular functions, cellular locations, or biological processes are arranged from the general to the specific, and these categories represent a universal way to describe gene function. Gene Ontology annotation results in a large amount of data that is difficult to synthesize manually, precluding quick insights into the results of upstream applications. Here, Sessa et al. (2023) describe and test GOgetter, an easy‐to‐use pipeline for the summarization and visualization of GO annotations from a set of FASTA files and a GO slim mapping file as input. GOgetter combines functionalities for transferring annotations via homology searchers, calculating summaries for every data set, and producing publication‐ready graphs. GOgetter is flexible, allowing users to apply different quality and similarity filters as well as use different reference databases to accommodate non‐model organisms. Three case studies demonstrate GOgetter's flexibility, wide applicability from bryophytes to angiosperms, and robustness. We anticipate that this software will facilitate the rapid exploration of new transcriptomes and genomes by streamlining the GO annotation process.

Bioinformatics has revolutionized plant biology, enabling researchers to harness analytical advancements and reveal the enormous complexity of plant genomes, relationships, and biology. As technological innovations promise to provide us with ever greater insights, our bioinformatic analyses of novel data types must keep pace by supporting techniques to further our understanding of plant biology, benchmarking methods for complex bioinformatic operations such as genome annotation, and contextualizing biological data in functional or structural terms. This special issue reflects the diversity of approaches to new and old problems in plant biology, showcasing the wide range of applications of bioinformatics in plant biology, and we hope that it will support the continuing development of bioinformatics tools and methods for a new generation of technological advance.

## AUTHOR CONTRIBUTIONS

K.E. prepared the initial draft of the manuscript. All authors contributed to article summaries, reviewed and edited subsequent drafts, and approved the final version of the manuscript.
